# Protandim, a Fundamentally New Antioxidant Approach in Chemoprevention Using Mouse Two-Stage Skin Carcinogenesis as a Model

**DOI:** 10.1371/journal.pone.0005284

**Published:** 2009-04-22

**Authors:** Jianfeng Liu, Xin Gu, Delira Robbins, Guohong Li, Runhua Shi, Joe M. McCord, Yunfeng Zhao

**Affiliations:** 1 Department of Pharmacology, Toxicology & Neuroscience, Louisiana State University Health Sciences Center, Shreveport, Louisiana, United States of America; 2 Department of Pathology, Louisiana State University Health Sciences Center, Shreveport, Louisiana, United States of America; 3 Department of Neurosurgery, Louisiana State University Health Sciences Center, Shreveport, Louisiana, United States of America; 4 Feist-Weiller Cancer Center, Louisiana State University Health Sciences Center, Shreveport, Louisiana, United States of America; 5 Department of Medicine, University of Colorado Health Sciences Center, Denver, Colorado, United States of America; Cleveland Clinic, United States of America

## Abstract

Oxidative stress is an important contributor to cancer development. Consistent with that, antioxidant enzymes have been demonstrated to suppress tumorigenesis when being elevated both in vitro and in vivo, making induction of these enzymes a more potent approach for cancer prevention. Protandim, a well-defined combination of widely studied medicinal plants, has been shown to induce superoxide dismutase (SOD) and catalase activities and reduce superoxide generation and lipid peroxidation in healthy human subjects. To investigate whether Protandim can suppress tumor formation by a dietary approach, a two-stage mouse skin carcinogenesis study was performed. At the end of the study, the mice on a Protandim-containing basal diet had similar body weight compared with those on the basal diet, which indicated no overt toxicity by Protandim. After three weeks on the diets, there was a significant increase in the expression levels of SOD and catalase, in addition to the increases in SOD activities. Importantly, at the end of the carcinogenesis study, both skin tumor incidence and multiplicity were reduced in the mice on the Protandim diet by 33% and 57% respectively, compared with those on basal diet. Biochemical and histological studies revealed that the Protandim diet suppressed tumor promoter-induced oxidative stress (evidenced by reduction of protein carbonyl levels), cell proliferation (evidenced by reduction of skin hyperplasia and suppression of PKC/JNK/Jun pathway), and inflammation (evidenced by reduction of ICAM-1/VCAM-1 expression, NF-κB binding activity, and nuclear p65/p50 levels). Overall, induction of antioxidant enzymes by Protandim may serve as a practical and potent approach for cancer prevention.

## Introduction

Cancer is affected by alterations in multiple physiological events including apoptosis, inflammation, differentiation, and angiogenesis. Oxidative stress, resulting from the imbalance between antioxidants and prooxidants, has been recognized to play an important role in cancer development. Consistent with that, antioxidant enzymes, especially superoxide dismutase (SOD), have been demonstrated to reduce tumorigenesis both *in vitro* and *in vivo* [1]–[2].

SOD not only suppresses cell proliferation, but also affects inflammation. The association between chronic inflammation and cancer is now well established [3]–[7]. One important mechanism of inflammation-induced cancer is due to oxidative stress [8]–[11], which results from the release of free radicals from activated immune cells and cytokines.

A new approach to antioxidant therapy via the induction of antioxidant enzymes (including SOD and catalase) has been developed [12]. The dietary supplement, Protandim, is a combination of five phytochemicals from medicinal plants long recorded in traditional Indian and Chinese medicine. One capsule of Protandim (675 mg) consists of the following: *B. monnieri* (45% bacosides), 150 mg; *S. marianum* (70–80% silymarin), 225mg; *W. somnifera* (1.5% withanolides), 150 mg; *C. sinensis* (98% polyphenols and 45% (-)-epigallocatechin-3-gallate), 75 mg; and *C. longa* (95% curcumin), 75 mg [12]. Protandim has been tested in healthy human subjects, producing increased SOD and catalase activities and decreased lipid oxidation levels in the blood samples without causing overt toxicity [12]. Being a critical step, studies have been performed to optimize the dosing of Protandim. At the current dose, none of the five ingredients alone significantly induces antioxidant enzymes; however, subtraction of any ingredient significantly reduces the induction. In summary, the bioactivities of each ingredient form a synergistic effect when combined [13].

In this study, the cancer preventative activity of Protandim is tested using a well-established two-stage skin carcinogenesis mouse model. The mouse skin carcinogenesis is a well-developed model to screen anti-cancer reagents. In this model, a tumor initiator (e.g., dimethylbenz[*a*]anthracene [DMBA]) is first treated to cause mutations of the oncogene Ras. A tumor promoter (e.g., 12-*O*-tetradecanoylphorbol-13-acetate [TPA]) is then applied to selectively promote the growth of Ras-mutated skin epidermal cells. As a phorbor ester, TPA can directly activate protein kinase C [14]. However, low concentrations of TPA can also exert inhibitory effect on cell proliferation either alone or in combination with anti-cancer drugs in human pancreas cancer cells [15], and human prostate cancer cells [16], [17]. A phase I dose escalation trial of TPA has been performed in patients with relapsed or refractory malignancies and the tolerated dose has been established [18].

## Materials and Methods

### Animals and treatment

#### For the two-stage skin carcinogenesis study

Six-to-eight-week-old female DBA/2 mice were purchased from the Jackson Laboratory (Indianapolis, IN) and housed in the LSUHSC-S Animal Resource Facility (four in one cage in microisolators) under standard regulations. The LSUHSC-S Animal Facility is AAALAC approved and maintains a consultation team of two veterinarians. The program is also monitored by the National Institute of Health Office for Protection from Research Risk and the U.S. Department of Agriculture. All animals were provided food and water ad libitum. Animals were euthanized under general anesthesia (overdose of pentobarbital) at the termination of each study. This method is consistent with the recommendation by the Panel of Euthanasia of the American Veterinary Medical Association.

These mice were divided into four groups: Basal diet (AIN-76A)+vehicle control (DMSO) group (5 mice); Protandim-containing basal diet+vehicle control group (5 mice); basal diet+DMBA+TPA group (15 mice); and Protandim-containing basal diet+DMBA+TPA group (15 mice).

A single dose of 100 nmol dimethylbenz[*a*]anthracene (DMBA) (Sigma, St. Louis, MO) dissolved in dimethylsulfoxide (DMSO, Sigma) was painted on the back of mice. After two weeks, mice were fed with either Protandim-containing diet (600 mg/kg diet; Protandim in power form was provided by Dr. Joe McCord) or basal diet (AIN-76A, both diets were manufactured by Dyets Inc [Bethlehem, PA]) till the end of the treatment protocol. After another two weeks, 4 µg of 12-*O*-tetradecanoylphorbol-13-acetate (TPA, Sigma), also dissolved in DMSO, was applied to the same area 5 days per week for 14 weeks. At the end of the treatments, the mice were euthanized and skin tissues were removed. Non-tumor skin tissues were carefully collected for biochemical and histological studies. To monitor the possibility of mycoplasma infection in the mouse skin tissues, the levels of mycoplasma pathogens were detected in a variety of tissue lysate (e.g., whole cell lysate, nuclear extract) using a MycoAlert Mycoplasma Detection Kit purchased from Lonza (Rockland, ME), and the results were negative. The remaining tumor-bearing tissues were fixed in 4% neutral buffered formaldehyde for subsequent pathological examination, which was performed by Dr. Xin Gu, a board-certified pathologist at LSUHSC-Shreveport.

#### For the in vivo SOD induction study

Twenty mice (8–10 weeks old, female, in DBA2 background) were divided into two groups (ten per group), fed with either basal diet or Protandim diet for three weeks. The mice were then euthanized, and skin epidermal tissues were collected. The total cell lysate was prepared using 50 mM phosphate buffer (pH 7.8), which was used to measure SOD expression/activity and catalase expression levels. The data was presented as mean±SD.

### Preparation of nuclear extract from skin tissues

At the end of the two-stage skin carcinogenesis study, non-tumor skin tissues were collected, and skin epidermal cells were stripped off as previously described [19]. Cells were suspended in 800 µL of buffer A (10 mmol/L HEPES-KOH with 1.5 mmol/L MgCl_2_, 10 mmol/L KCl, 0.2 mmol/L phenylmethylsulfonyl fluoride, and 5 µmol/L DTT) containing protease inhibitors (5 µg/mL each of pepstatin, leupeptin, and aprotinin), and homogenized in a 10-mL Wheaton homogenizer. After a short (10-second) spin, the supernatant was kept on ice for 30 minutes, 25 µL of 10% NP40 was then added, and the sample was vortexed vigorously for 25 seconds. The lysate was centrifuged at 17,000×*g* for 1 minute. The resulting pellet was dissolved in 120 µL of buffer B [20 mmol/L HEPES-KOH with 1.5 mmol/L MgCl_2_, 420 mmol/L NaCl, 35% glycerol, 0.2 mmol/L phenylmethylsulfonyl fluoride, 5 µM of DTT, and 0.2 mmol/L EDTA (pH 8.0)] containing the above protease inhibitors. The sample was kept on ice for 30 minutes, followed by centrifugation at 14,000×*g* for 5 minutes, and the supernatant, identified as *nuclear extract*, was frozen at −80°C.

### Preparation of whole cell lysate from skin tissue

Skin epidermal cells, stripped from non-tumor tissues, were extracted by homogenization in 1 mL of Homogenization Buffer [20 mmol/L HEPES (pH 7.0), 5 mmol/L EGTA, 10 mmol/L 2-mercaptoethanol, 1 mmol/L phenylmethylsulfonyl fluoride, and 1 µg/mL each of the protein inhibitors mentioned above]. The lysate was centrifuged (50×*g* or 600 rpm, 5 minutes) to remove tissue debris. The resulting supernatant was centrifuged at 10,000×*g* for 1 hour at 4°C. The supernatant was transferred into a new tube and designated as *whole cell lysate* and kept at −80°C.

### MnSOD and total SOD activity assays

The MnSOD and total SOD activities were measured using the NBT-BCS SOD inhibition assay as previously described by Spitz and Oberley [20]. Total cell lysate was prepared similarly to that of whole cell lysate except that 50 mM phosphate buffer (pH 7.8, containing proteinase inhibitors) was used to replace the Homogenization Buffer. The assay buffer contained xanthine-xanthine oxidase which generated superoxide; NBT was then reduced by superoxide to form blue formazan. The presence of SOD inhibited NBT reduction. The data was plotted as percentage inhibition vs. protein concentration. One unit of activity was defined as the amount of protein needed to inhibit 50% of the NBT reduction rate. NaCN (5 mM) was used to measure MnSOD activity and the CuZnSOD activity was obtained after subtracting the MnSOD activity from the total SOD activity. BCS was used to inhibit tissue specific interferences.

### Detection of oxidatively modified proteins (protein carbonyls)

The OxyBlot protein oxidation detection kit (s7150, Intergen, Purchase, NY) was used to perform the assay. The reaction procedures were conducted according to the manufacturer's instructions. Ten percent SDS-PAGE gels were used for the separation of *whole cell lysate* of the stripped skin epidermal cells.

### H&E staining of the skin tissues

At the end of the skin carcinogenesis study, 3 small pieces (approximately 4 mm^3^ each) of non-tumor skin tissues were fixed in 4% neutral buffered formaldehyde for the following histological studies. Tissue embedding, processing, and Hematoxylin & Eosin (H&E) staining (to detect skin hyperplasia) were carried out at the Research Core Laboratory at the Department of Anatomy and Cell Biology of LSU Health Sciences Center-Shreveport, following the standard laboratory protocols.

### Macrophage staining of the skin tissues

Slides with non-staining tissues were deparafinized followed by antigen retrieval using a steamer. The slides were then pre-treated with 0.5% peroxide followed by blocking with 10% normal goat serum. A rat anti-mouse F4/80 antibody (Invitrogen, Carlsbad, CA; 1∶100 dillution in 5% normal serum) was used to stain macrophages, and rat serum was used for blocking. The samples were then stained using an ABC staining system following the procedures provided by the manufacturer (Santa Cruz). Finally, the samples were counterstained with hematoxylin. Graphs were randomly taken from the stained slides using a Zeiss Axiophot photomicroscope (amplification: 20×).

### Electrophoretic mobility shift assays (EMSAs)

Nuclear factor-κB (NF-κB)-DNA binding activity was analyzed using *nuclear extract*. The NF-κB double-strand oligonucleotide (5′-AGTTGAGGGGACTTTCCCAGGC-3′) was purchased from Promega (Madison, WI). The 25-µl reaction solution contained 6 µg of *nuclear extract*, 5 µl of 5× binding buffer (50 mM Tris-HCl, pH 7.4, with 20% glycerol, 5 mM MgCl_2_, 2.5 mM EDTA, 5 mM DTT, and 0.25 mg/ml poly dI-dC) and 50,000 cpm labeled probe. After 20 minutes of incubation at room temperature, 3 µl of 10× loading buffer was added, and samples were separated on a 6% native polyacrylamide gel for 3 to 4 hours. The gel was dried and DNA-protein complexes were visualized by exposing the gel to a Kodak film at −80°C. A supershift assay was performed in a separate gel, following the procedures similarly to what has been described [21] with slight modifications [22]. The anti-p65 or p50 antibodies for supershift (10×, purchased from Santa Cruz) were incubated with *nuclear extract for 1 h at room temperature before addition of the labeled probe*.

### Western Blot analysis

*Whole cell lysate* was used to detect the expression levels of (p)PKCε, JNK, pJNK, ICAM-1, VCAM-1, and GAPDH. *Nuclear extract* were used to detect the expression levels of Jun D, Fra-1, NFκB subunit p65 and p50, and Lamin B. Twenty micrograms of w*hole cell lysate* or 5 micrograms of *nuclear extract* was separated on a 10% SDS-PAGE gel and transferred onto a PVDF membrane. Ponceau S staining was used to monitor uniform transfer of protein. All primary and secondary antibodies were purchased from Santa Cruz Biotechnology (Santa Cruz, CA). The antibody bands were visualized by the enhanced chemiluminescence detection system (ECL, Amersham Pharmacia Biotech, Piscataway, NJ). The membranes were then stripped and reprobed with an anti-GAPDH or anti-Lamin B antibody to normalize protein loading. The corresponding bands were scanned, and the density was quantitatively assessed using VersaDoc Imaging System equipped with QuantityOne Software (Bio-Rad, Hercules, CA).

There were five tissues samples in each group and the Western blot results were semi-quantitatively analyzed as the following: the density of each band of interest was divided by the density of the corresponding loading control band. For comparisons, the Basal diet/Vehicle was set up as the control group, and the ratios were calculated based on this group.

### Statistical analysis

Statistical analysis was performed using both a Student's t-Test (with two-tailed distribution and two-sample equal variance) (for two-group comparison) and one-way ANOVA (for multiple-group comparison) followed by the Newman-Keuls post-test. p<0.05 was judged to be significantly different.

## Results

### Protandim diet suppressed skin tumor formation

We first tested whether the Protandim diet suppresses tumor formation in a two-stage skin carcinogenesis model. At the end of the study, no significant difference in the body weight between the basal diet group (Basal diet/DMBA+TPA, n = 15) and the Protandim diet group (Protandim/DMBA+TPA, n = 15) indicated no overt toxicity (26.5±1.3 g vs 26.0±1.0 g, p = 1.3). The pathological examination is summarized in Table 1. The results show that there was no tumor formation in the two (basal diet and Protandim diet) vehicle control groups. In the two DMBA+TPA treatment groups, 100% of the mice from the basal diet group developed tumors, and the average number of tumors per mouse in this group was 6.3. Only 66.7% of the mice from the Protandim diet group developed tumors, and the average number of tumors per mouse was 2.7. These results revealed that the Protandim diet reduced tumor incidence by 33% and multiplicity by 57% (p = 0.003).

**Table 1:** Papilloma formation in the multistage carcinogenesis model.

Treatment	Number of Mice	Tumor Incidence	Papillomas/mouse	Total Papillomas
Basal diet/TPA	15	100%	6.3±3.3	94
Protandim/TPA	15	66.7%	2.7±2.7*	40
Basal diet/Vehicle	5	0%	0±0	0
Protandim/Vehicle	5	0%	0±0	0

* p = 0.003 compared with the basal diet/TPA group. Vehicle: DMSO.

### Protandim diet suppressed TPA-induced cell proliferation

**Figure 1:**
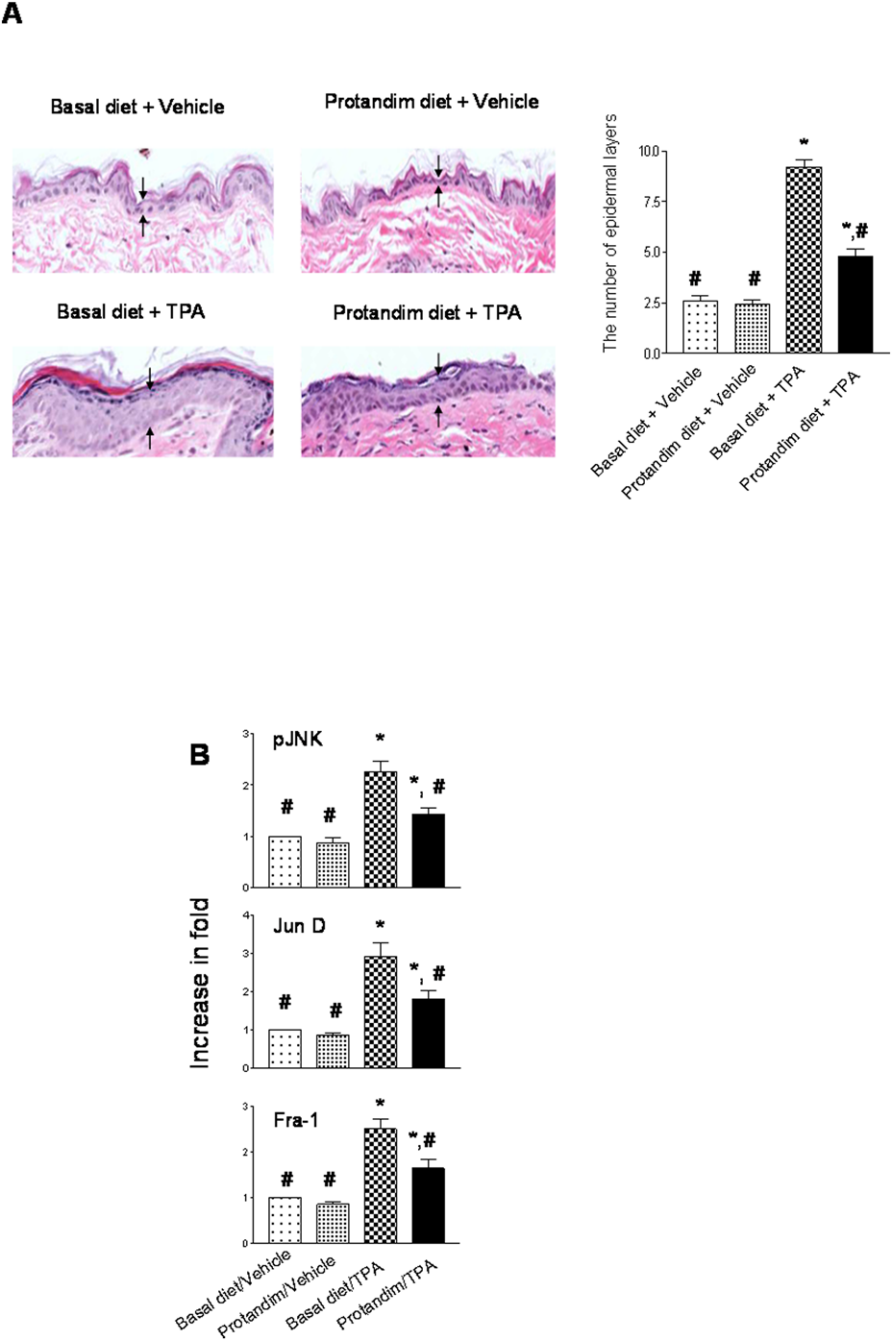
Detection of cell proliferation markers contributing to skin carcinogenesis. Samples were collected at the end of the skin carcinogenesis study (n = 5 per group). Data was reported as means±standard error (SEM), and p<0.05 was judged to be significantly different. *, significantly different from the basal diet/Vehicle group; #, significantly different from the basal diet/TPA group. Vehicle: DMSO. (A) H&E staining of skin epidermal tissues. A representative result was shown. Arrows point to the layer of skin epidermis. The histologic evaluation was conducted by a pathologist (X.G.) who had no knowledge of the treatments. The number of skin epidermal layers was plotted and statistical analysis was performed using one-way ANOVA followed by the Newman-Keuls post-test. (B) Semi-quantitative analysis of the protein levels of pJNK, Jun D, and Fra-1. Vehicle: DMSO. The levels of pJNK were normalized to that of JNK, and the levels of Jun D and Fra-1 were normalized to that of Lamin B. Statistical analysis was performed using one-way ANOVA (for multiple-group comparison) followed by Newman-Keuls post-test.

Skin epidermal hyperplasia was revealed in H&E stained tissues. As shown in Figure 1A, TPA treatment induced cutaneous hyper-proliferation in the basal diet group, whereas Protandim diet reduced the levels of hyperplasia.

To detect the effect of the Protandim diet on the signaling molecules contributing to tumor promotion, non-tumor tissues were collected at the end of the skin carcinogenesis study, and Western blot analysis was performed. As the important oncoproteins and subunits of the transcription factor - activator protein- 1 (AP-1) in skin carcinogenesis, Jun and Fos family members have been previously studied [23]. The results demonstrate that Jun D and Fra-I make major contributions to AP-1 binding activity [23]. As summarized in Figure 1B, in the samples from the basal diet group, TPA treatment induced a 2.8-fold increase in Jun D expression and a 2.5-fold increase in Fra-1 expression in the nucleus, compared to a 1.7-fold increase in Jun D and a 1.6-fold increase in Fra-1 in the Protandim diet group. The expression levels of phosphorylated c-Jun N-Terminal Kinase (pJNK) were next examined using w*hole cell lysate*. Also in Figure 1B, in the samples from the basal diet group, TPA treatment induced a 2.3-fold increase in pJNK protein levels (normalized to the total levels of JNK), compared to a 1.4-fold increase in the Protandim diet group. Protandim diet alone did not affect either Jun D/Fra-1 or pJNK level significantly.

### Protandim diet suppressed TPA-induced cutaneous inflammation

TPA is known to incite cutaneous inflammation, which further contributes to skin tumor formation [24]–[25]. To investigate whether the Protandim diet modulates cutaneous inflammation as a mechanism of its tumor suppressive effects, the same set of tissues from the cell proliferation study was also used for the following study.

**Figure 2:**
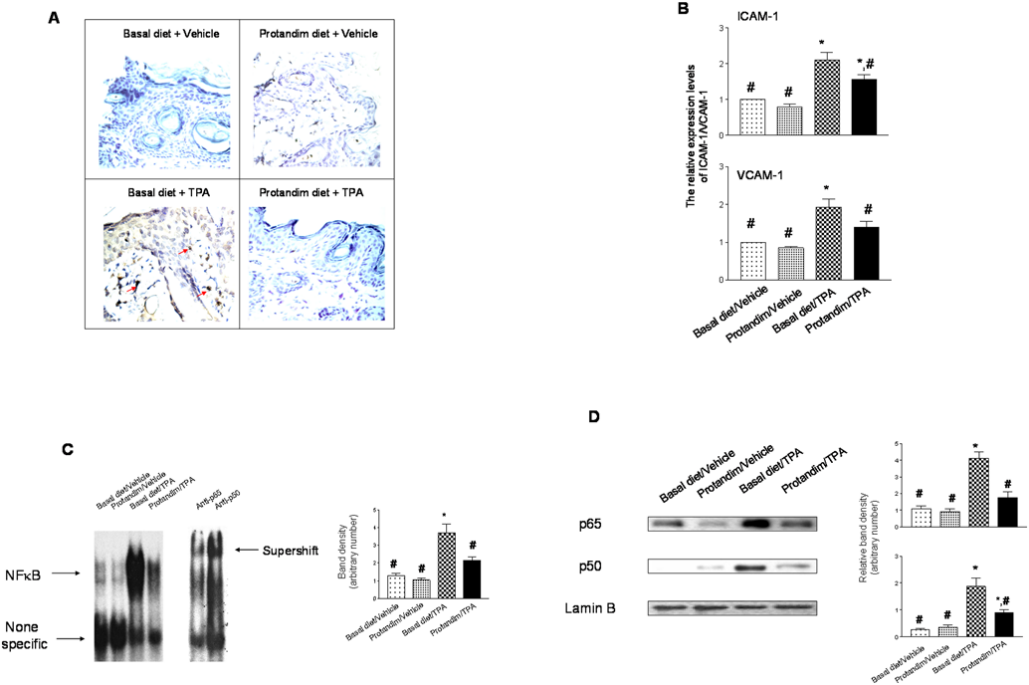
Detection of cutanous inflammation markers. Samples were collected at the end of the skin carcinogenesis study (n = 5 per group). (A) Immunohistochemical staining of macrophages in mouse skin tissues. The skin samples were prepared at the end of the two-stage carcinogenesis study. The arrows (in red) indicate macrophage staining. Amplification: 20×. (B) Semi-quantitative analysis of the protein levels of ICAM-1 and VCAM-1. The levels of ICAM-1/VCAM-1 were normalized to that of GAPDH. Statistical analysis was performed using one-way ANOVA (for multiple-group comparison) followed by Newman-Keuls post-test. Data was reported as means±standard error (SEM), and p<0.05 was judged to be significantly different. *, significantly different from the basal diet/Vehicle group; #, significantly different from the basal diet/TPA group. Vehicle: DMSO. (C) Electrophoretic mobility shift analysis of NF-κB-DNA binding activity. A representative result was shown. The specific bands were quantified and plotted, and statistical analysis was performed using one-way ANOVA. For the supershift experiment, either anti-p65 or anti-p50 antibody (both are concentrated antibodies) was preincubated with *nuclear axtract* for 1 h at room temperature before addition of the labeled probe. The Basal diet/TPA samples were chosen for the assay. Vehicle: DMSO. (D) Western blot analysis of the nuclear levels of p65 and p50. Lamin B served as the loading control. A representative result was shown. The p65/p50 bands were quantified and normalized to the corresponding Lamin B bands. Statistical analysis was performed using one-way ANOVA. Vehicle: DMSO.

Immunohistochemical examination of infiltrated macrophage was first detected in the skin tissues. As shown in Figure 2A, in vehicle (DMSO) treated mice, whether on Basal diet or Protandim diet, few macrophages were observed in the mouse skin tissues. There were increased infiltrated macrophages in TPA-treated Basal diet group; whereas less macrophages were observed in TPA-treated Protandim diet group.

Western blot analysis was performed to detect the expression of ICAM-1 and VCAM-1 in skin epidermal cells using *whole cell lysate*. As shown in Figure 2B, both ICAM-1 and VCAM-1 levels were significantly increased (2-fold) upon TPA treatment in the samples from the basal diet group. These increases were significantly reduced in the samples from the Protanidm diet group.

NF-κB is a pivotal regulator of proinflammatory gene expression [26]. The NF-κB–DNA binding activity was detected using EMSA. As shown in Figure 2C, TPA treatment induced increases in NF-κB–DNA binding activity in the samples from the basal diet group, whereas these increases were reduced in samples from the Protandim diet group. The expression levels of p65 and p50, as two subunits of NF-κB, were also detected using *nuclear extract* (Figure 2D). Consistent with the increases in NF-κB binding activity, the protein levels of the two subunits were also increased upon TPA treatment in the samples from the basal diet group, and these increases were reduced in the Protandim diet group.

### Protandim diet induced superoxide dismutase (SOD) and catalase, leading to suppression of oxidative stress in skin epidermal tissues

**Figure 3:**
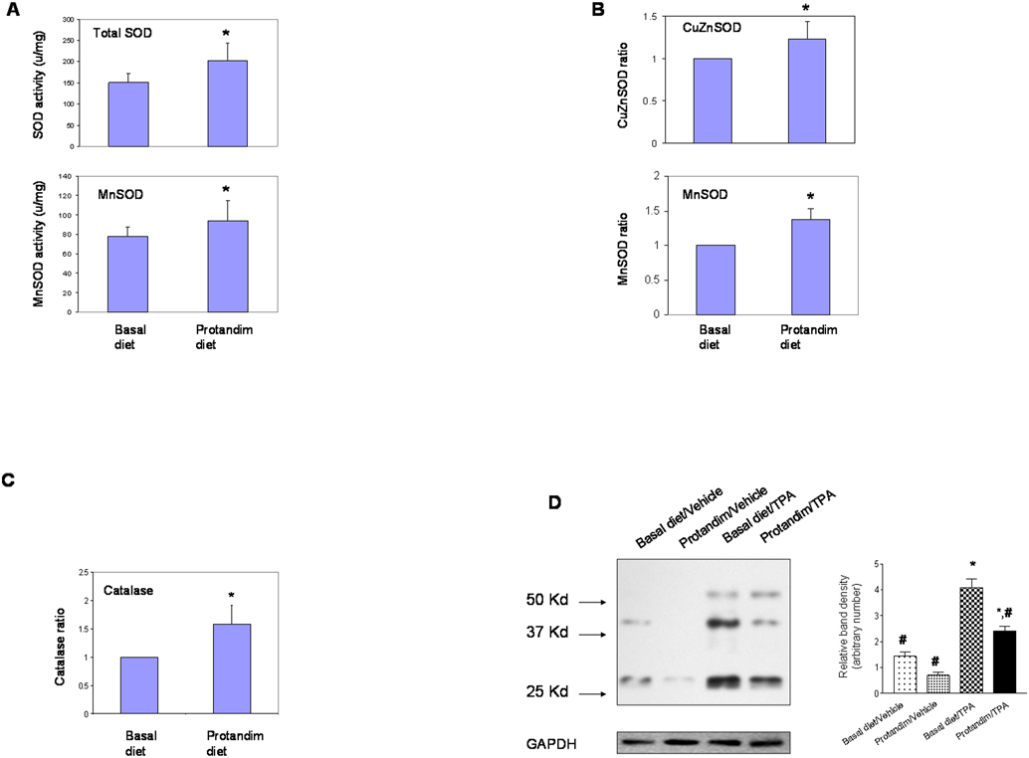
Detection of oxidative stress and antioxidant enzymes in skin epidermal tissues. For Figure 3A to 3C, skin tissues from the three-week dietary supplementation study were used (n = 10 per group). Statistical analysis was performed using Student's t-test. Data was reported as means±standard deviation (SD), and p<0.05 was judged to be significantly different. *, significantly different from the basal diet group. For Figure 3D, skin tissues from the two-stage skin carcinogenesis study were chosen (n = 5 per group). (A) Total SOD and MnSOD activity. For the total SOD activity, the numbers were 150.3±22.3 (mean±S.D.) vs 202.8±41.0, and p = 0.0039. For the MnSOD activity, the numbers were 77.7±9.8 vs 94.3±20.1, and p = 0.039. (B) Semi-quantitative analysis of the protein levels of MnSOD and CuZnSOD. (C) Semi-quantitative analysis of the protein levels of catalase. (D) Detection of oxidatively modified proteins. A representative result was shown. All of the carbonyl-modified bands in each lane were quantified, combined, and normalized to GAPDH. Statistical analysis was performed using one-way ANOVA (for multiple-group comparison) followed by Newman-Keuls post-test. Data was reported as means±standard error (SEM), and p<0.05 was judged to be significantly different. *, significantly different from the basal diet/Vehicle group; #, significantly different from the basal diet/TPA group. *Vehicle: DMSO.*

Protandim is designed to induce primary antioxidant enzymes, which was first demonstrated in healthy human subjects [12]. In order to determine whether the Protandim diet could induce SOD expression/activity and catalase expression in skin epidermal tissues within a short period of time, animals were fed with either of the two diets for three weeks. Total cell lysate from the collected skin epidermal tissues was used to detect the SOD expression/activity levels, and the results are summarized in Figure 3. In detail, there was a 35% increase in total SOD activity and a 21% increase in MnSOD activity in the Protandim diet group (Figure 3A), which is consistent with the results seen in the human studies [12]. The increases in SOD activities are likely caused by the induction of protein expression, since there was a 37% increase in MnSOD and a 23% increase in CuZnSOD expression levels (Figure 3B). The expression levels of catalase were also detected, and there was a 58% increase in the Protandim diet group (Figure 3C).

Finally the levels of protein carbonyls, as an oxidative stress marker, were detected. As shown in Figure 3D, TPA treatment induced increases in the oxidatively modified proteins at the molecular weight 54, 42, and 27 kd in the samples from the basal diet group. These increases were reduced in samples from the Protandim diet group.

## Discussion

Oxidative stress plays a positive role during cancer development. Overexpression of antioxidant enzymes, especially manganese superoxide dismutase (MnSOD), has been demonstrated to suppress tumorigenesis when being elevated both *in vitro* and *in vivo* [2]. To seek a more practical antioxidant approach which can be used for dietary based chemoprevention, Protandim, a combination of five medicinal plants has been developed and a preliminary test in humans has been performed [12]. Each ingredient of Protandim has shown antioxidant activities (Bacoside [27]; silymarin [28]; W. somnifera powder [29]; green tea [30]; curcumin [31]). However, the combination of ingredients creates a synergistic effect and lowers the concentration of each ingredient in the induction of Nrf2-regulated antioxidant enzymes [13]. While CuZnSOD, MnSOD, and catalase are probably not directly upregulated by Nrf2 (as they do not appear to contain Antioxidant Response Elements in their promoters), they have been reported to be included in the array of enzymes induced by Nrf2 activators. MnSOD, e.g., has been shown to behave like heme oxygenase-1 and glutamate cysteine ligase (both of which are directly Nrf2-regulated) with regard to silencing of Nrf2 via siRNA knockdown studies [32]. One possible mechanism for “secondary” induction of MnSOD by Nrf2 activation is the upregulation and nuclear translocation of thioredoxin by Nrf2 [33]. Thioredoxin, itself, is a transcription factor that has been shown to induce MnSOD [34].

We first tested the chemo-preventative effect of Protandim using a well-developed skin carcinogenesis mouse model. The human dose of Protandim of 675 mg/d is approximately 10 mg/kg/d. A 20-g mouse should receive nearly 2.5 mg/d to receive an equivalent dose, according to the equation established by Reagan-Shaw et al [35]. The amount of Protandim per kg chow we used (600 mg/kg) delivers almost exactly 2.5 mg of Protandim if the mouse eats a little over 4 g of chow per day. So we chose a dose equivalent to the human dose (taking one Protandim caplet per day). Within three weeks, SOD activity/expression levels were induced by Protandim diet. The increased level in MnSOD activity (21%) is less than that in MnSOD expression (37%). This might be due to the interaction between MnSOD and p53, which leads to inactivation of MnSOD activity, as previous studies have demonstrated [36]. Protandim is a combination of five ingredients designed to induce SOD and catalase. It is likely that this combination will also induce many additional antioxidant enzymes that are Nrf2-dependent, whether directly or indirectly. It has recently been shown that Protandim causes the translocation of Nrf2 to the nucleus [13]. Indeed, the expression levels of heme oxygenase-1 (HO-1, data not shown) were also increased in the same set of samples for SOD detection. MnSOD became the major interest for this study, based on the previous skin carcinogenesis studies which show that only overexpression of MnSOD suppresses skin tumor formation, but not overexpression of CuZnSOD [37], nor glutathione peroxidase [37]. To our knowledge, no studies using HO-1 or catalase overexpression mice in skin carcinogenesis have been reported.

Oxidative stress contributes to multiple physiological events including cell proliferation and inflammation, mediated by modifying redox sensitive AP-1 (proliferation) and NF-κB (inflammation) pathways. The nature of mitogen (e.g. TPA)-induced inflammation includes the generation of superoxide radicals. Superoxide radicals could serve as signaling molecules to induce cell proliferation. TPA can also directly bind to PKC; members of the PKC family regulate downstream of nearly all membrane-associated signal transduction pathways. In addition to promoting cell proliferation, in PKCα-overexpressed primary keratinocytes, NF-κB is a pivotal regulator of proinflammatory gene expression [25]. Furthermore, TNFα is PKC-inducible when overexpressed in mouse keratinocytes, and is highly induced in PKC transgenic skin [38]. These studies clearly demonstrate that cell proliferation and inflammation can indeed interact during skin carcinogenesis. Since the NF-κB pathway has an essential role in adaptive response and cell survival [39], increasing evidence suggests that deregulation of NF-κB and its regulatory kinases play a contributing role to cancer development, progression, and resistance to chemotherapy [40]–[41]. Thereafter, developing inhibitors of the NF-κB pathway might potentiate the therapeutic effects of chemo drugs, through the mechanism of enhancing cell death [42]. Such efforts have been illustrated in a recent study by Bauer et al. [43], the authors showed enhanced drug efficacy using nitrosylcobalamin, which inhibits NF-κB activation and its survival signaling.

Each of the five ingredients of Protandim shows anti-cancer effects and other activities. Bacosides, a traditional Ayurvedic medicine, has been used in India for centuries as a memory enhancing, anti-inflammatory, analgesic, antipyretic, sedative and antiepileptic agent [44]. Silymarin is known as a hepatoprotectant, but also shows anti-cancer and cytoprotective activities on organs including the prostate, lungs, CNS, kidneys, pancreas and skin [45]. W. somnifera has shown anti-angiogenesis and anti-cancer activities [46]–[47]. Green tea (EGCG) shows promising results in cancer prevention and treatment in a large number of studies [48]–[52]. Curcumin is another rising star as a cancer prevention agent [53]–[56]. However, the benefits of forming this combination include: 1) existing a synergistic effect; and 2) lowering the concentration of each ingredient to reduce the potential side effects.

In summary, tumor promoter TPA incites cutaneous proliferation and inflammation mediated at least in part, by oxidative stress. Protandim prevents skin tumor formation via the induction of several primary antioxidant enzymes. As the induction of antioxidant enzymes is a much more potent approach than supplementation with conventional stoichiometric antioxidants, Protandim may be suitable for translational research and may serve as a therapeutic approach for cancer prevention.
